# Large‐Scale Genomic Surveillance of Swine Influenza A Viruses in Europe Highlights Increased Reassortment Potential and Field‐Based Associations With Clinical Manifestations

**DOI:** 10.1155/tbed/7198173

**Published:** 2026-11-01

**Authors:** Kathrin Lillie-Jaschniski, Zalán Homonnay, Gwenaëlle Dauphin, István Kiss, Susanne Zoels, Julia Stadler, Nick Vereecke

**Affiliations:** ^1^ Ceva Animal Health, Libourne France; ^2^ Scientific Support and Investigation Unit Ceva-Phylaxia Campus Budapest Hungary; ^3^ Clinic for Swine Centre for Clinical Veterinary Medicine Ludwig-Maximilians-Universität München Oberschleissheim Germany uni-muenchen.de; ^4^ Laboratory of Virology Department of Translational Physiology Infectiology and Public Health Faculty of Veterinary Medicine Ghent University Merelbeke Belgium ugent.be; ^5^ PathoSense BV Lier Belgium

**Keywords:** cross-associations, One Health, reassortment dynamics, swine influenza virus

## Abstract

Swine influenza A virus (swIAV) is an important One Health pathogen due to its genetic diversity, ongoing reassortment, and implications for animal and public health. Between 2022 and 2023, nasal swabs (NSs) from pigs of multiple age groups were collected from 134 farms across 16 European countries and analyzed by real‐time quantitative PCR (RT‐qPCR), subtyping, and whole‐genome sequencing. Clinical and management metadata were integrated with viral genotypes. swIAV was detected in 93% of the targeted farms included in this study, with Clade 1A (H1pdmN1) and Clade 1C (H1avN1, H1avN2, and H1huN2) predominating. Sequencing identified 23 genotypes, including 12 previously unreported ones. Exploratory analysis suggested potential associations between specific hemagglutinin (HA) (sub)clades and clinical signs in piglets and with herd management factors. High genotype diversity—especially in Spain (ES)—suggests potential associations with intensive production systems. Overall, this large European survey indicates frequent circulation of swIAV within the targeted study population, accompanied by high genetic diversity and ongoing reassortment, underscoring the need for harmonized genomic surveillance to inform veterinary and public health preparedness.

## 1. Introduction

Swine influenza A virus (swIAV) is a key component of the porcine respiratory disease complex and can also impair reproductive performance [1, 2]. Subclinical infections further complicate efforts to control the virus by facilitating persistent transmission [3–5]. Given its zoonotic potential and ability to reassort with avian and human influenza viruses, continuous genomic surveillance is essential. The circulation of swIAV at the human–animal interface poses an ongoing zoonotic risk and represents a critical One Health challenge [6, 7]. Current diagnostic and surveillance approaches—whether based on targeted sampling of symptomatic pigs or cross‐sectional group sampling—are effective for detecting existing infections and identifying the swIAV subtype, (sub)clade, lineage, and/or genotype [8–10]. However, these methods typically do not provide insights into how specific viral strains are linked to clinical outcomes or management factors. Although numerous studies have used next‐ and third‐generation sequencing to describe the genetic diversity of swIAV, most of these investigations have focused solely on molecular characterization and phylogenetics [11–14]. To date, few efforts have been made to systematically link whole genome sequencing (WGS) (i.e., sequence information on each of the eight individual ssRNA segments) to clinical signs or herd‐level management practices under field conditions. As a result, the clinical relevance of many circulating genotypes remains uncertain, and the practical utility of WGS data in disease control remains limited. A better understanding of these connections could support more effective intervention strategies and inform herd‐level decision‐making. Therefore, this study aims to address this knowledge gap by exploring statistical associations between hemagglutinin (HA)‐ and neuraminidase (NA)‐segments derived from swIAV WGS data identified on 134 farms in 16 European countries and detailed clinical and herd management metadata. Our objective was to explore potential associations between viral genotypes (primarily focused on HA/NA (sub)clades), observed clinical signs across different age groups, and farm‐level factors, including biosecurity and production practices. This approach may not only leverage the value of continuous genomic surveillance but also provide new insights to improve vaccination strategies and optimize herd management.

## 2. Materials and Methods

### 2.1. Farm Selection, Sampling, and Metadata Collection

Between October 2022 and September 2023, a cross‐sectional sampling was conducted in 134 pig farms in 16 European countries (including Austria [AT], Belgium [BE], Bulgaria [BG], Denmark [DK], France [FR], Hungary [HU], Germany [DE], Greece [GR], Italy [IT], North Macedonia [MK], United Kingdom [UK], Poland [PL], Portugal [PT], Slovenia [SI], Spain [ES], and the Netherlands [NL], Figure 1). The study aimed to detect as many swIAV‐positive farms as possible and therefore focused on farms with a high likelihood of swIAV infection. Thus, the farms were selected based on the following inclusion criteria: (i) farms with either acute clinical signs suggesting swIAV infection, including dyspnea, coughing, sneezing, nasal discharge, anorexia, and/or lethargy (epidemically infected farms), (ii) farms with a known influenza history, or (iii) farms suspecting endemic swIAV circulation due to either reproductive failure and fever in sows or recurrent respiratory distress in the nursery (endemically infected farms). To achieve a minimum detection probability of 95% at an assumed prevalence of 15% (diagnostic sensitivity 90% and specificity 100%), approximately 30 pigs per farm were sampled across age groups. The sampling protocol was designed to include 10 nasal swabs (NSs) in each of the following age groups: suckling piglets (1–4 weeks of age [w.o.a.]), piglets around weaning (4–6 w.o.a.), and adult piglets. and animals in the middle/end of the nursery period (7–9 w.o.a.). In the suckling period, one piglet per litter was selected from a total of 10 litters. In piglets after weaning and in the middle/end of the nursery period, one piglet per pen from around 10 pens was sampled. Depending on the farm type, additional age groups—fattening pigs, gilts, and sows—were included following the same sampling approach used for nursery pigs (fatteners: 10 NS beginning fattening (14–16 w.o.a.) 10 NS middle/end fattening (20–22 w.o.a.), 10 NS of gilts, and 10 NS of sows). The collection of NS was performed by inserting the swab 2–4 cm into both nostrils of each animal and rotating it 360°. NSs were collected and investigated as pools (five individual swabs per tube), yielding a minimum of six pooled samples per farm. In total, 554 pooled samples were collected. Swabs were placed into a plastic tube with 2 mL of viral media (Virocult) and transported under cooled conditions to local laboratories for testing, and aliquots were stored at −70°C for downstream analysis.

**Figure 1:**
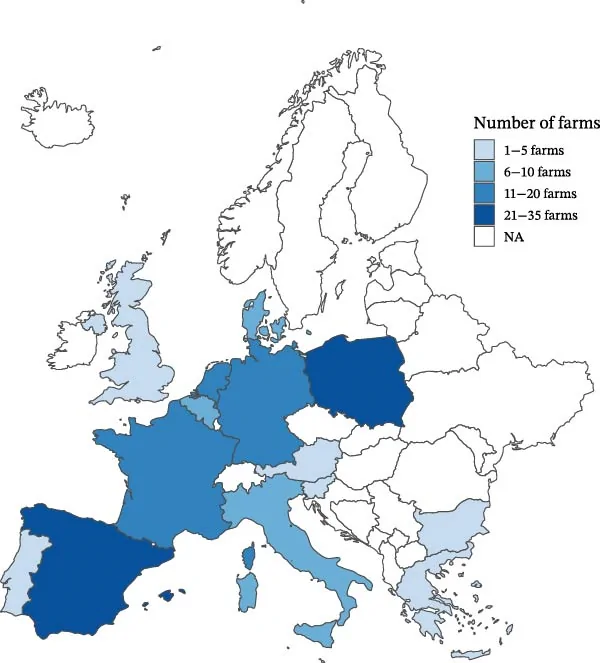
Map of Europe highlighting the 16 European countries that participated in the study. Each country is colored according to the number of sampled farms colored in shades of blue with increasing number of farms. European countries not included in the study are indicated with “not available (NA).”

An extensive and standardized metadata collection was applied and is summarized in Table S2, which contains both farm‐ and sample‐level metadata. This metadata collection included information on swIAV status of the herds (i.e., endemically or epidemically infected), the farm size (categorized into three groups, including 50–499 sows, 500–999 sows, and 1000–10,000 sows), swIAV vaccination status, and the clinical signs exhibited by the sampled animals were recorded (Table S5). Herd examination also included a questionnaire assessing the production and management parameters of the sampled age groups.

### 2.2. Real‐Time Quantitative PCR (RT‐qPCR) Screening and Subtyping

Each pool of NS was first investigated by RT‐qPCR targeting the matrix (M) gene of swIAV at a local laboratory. Results with a cycle threshold (Ct) value ≤ 39 were considered positive, and samples with Ct > 39 were considered negative. All positive samples with a Ct ≤ 30 were further investigated via multiplex RT‐qPCR to determine the swIAV subtype by HA and NA gene amplification, targeting H1av, H1pdm, H1hu, H3hu, and N1 (including N1all and N1pdm), and N2. If only the HA could be determined, samples were counted as typable. Both the swIAV RT‐qPCR and multiplex RT‐qPCR were performed according to the protocol of Henritzi et al. [15]. Original samples were retained for downstream analyses, and at the end of the study, all positive samples with a Ct‐value ≤ 30 were sent on dry ice to SSIU‐Ceva‐Phylaxia, Budapest, Hungary, for third‐generation long‐read nanopore sequencing (File S2).

### 2.3. Targeted swIAV Long‐Read Nanopore WGS

RNA was re‐extracted from original samples with the ZymoBIOMICS DNA/RNA Miniprep Kit (Zymo Research) according to manufacturer’s instructions, followed by the removal of (genomic) DNA using the Invitrogen ezDNase Enzyme (Thermo Fisher Scientific) according to manufacturer’s protocol. First, DNA‐free RNA was subjected to a swIAV RT‐qPCR to reconfirm positivity with the VetMAX‐Gold SIV Detection Kit (Thermo Fisher Scientific), followed by targeted amplification of swIAV genome segments using the Invitrogen SuperScript IV One‐Step RT‐PCR System (Thermo Fisher Scientific), using the “MBTuni primer set” as described by Zhou and Wentworth [16]. Quality control of the resulting PCR products was done using the E‐gel electrophoresis system (Thermo Fisher Scientific) with 2% SYBR safe agarose gels. Samples without expected bands were excluded from the sequencing. The cDNA amplicon concentrations were determined with the Qubit RNA HS Assay Kit on the Qubit Fluorometer (Thermo Fisher Scientific), and 200 ng of each sample was used for long‐read nanopore library preparation using the Native Barcoding Kit 24 V14 (SQK‐NBD114.24; Oxford Nanopore Technologies [ONT]), allowing the multiplexing of a maximum of 24 samples per sequencing run, according to manufacturer’s instructions. Sequencing was carried out on R10.4.1 flow cells on a MinION Mk1B portable sequencer (ONT). Data were base called in real‐time in MinKNOW (v. 24.06.16; ONT), applying Dorado (v.7.4.14) basecalling using the high accuracy model linked to demultiplexing and removal of adapters. Alignments were done using minimap2 within Geneious Prime. The consensus ORF sequences of the segments were aligned and clustered (neighbor‐joining method and Tamura‐Nei distance model) with reference sequences of HA/NA subtypes and genetic clades to determine their HA/NA subtype, (sub)clade, and lineage (given in curated dataset in Table S3). Final genome sequences were uploaded to GISAID under accessions EPI_ISL_20222101‐20222189, EPI_ISL_20222258‐20222259, and EPI_ISL_20222281.

### 2.4. Phylogenetic and Statistical Cross‐Association Analyses

The HA segment sequences were subjected to phylogenetic inferences using IQtree2 (v2.3.5; ‐m TEST ‐T AUTO ‐bb 100,000; [17]) on a multiple sequence alignment as obtained from MAFFT (v.7.526; [18]). For the classification of HA gene (sub)clades, the online tool of BV‐BRC (https://www.bv-brc.org/app/SubspeciesClassification) was used. The final phylogenetic tree summarized a total of 178 HA sequences, which were pruned by removing redundant data based on the following criteria: (i) only one representative per sampling date and farm ID, (ii) exclusion of mixed swIAV genotypes, showing more than one genotype in either NA or HA segments (e.g., N1 and N2 segment or 1C.2.2 and 1A.3.3.2 HA (sub)clades identified from sequencing data), and (iii) exclusion of samples with missing HA/NA sequences. This was important to ensure robust and unbiased cross‐association analysis and resulted in a final phylogenetic tree comprising 92 HA sequences. After the removal of redundant samples and harmonization of metadata, a curated dataset was generated for downstream phylogenetic and association analyses (Table S3). The phylogenetic tree was visualized and annotated with metadata in iTOL (v.7; [19] and Table S3). The resulting cleaned and extended metadata were subjected to an extended cross‐association analysis building further on preliminary findings as described in Stadler et al. [20]. A Python‐based approach included independent statistical assessments dependent on the compared variable types (continuous (i.e., numeric, ≥3 unique values), binary (i.e., exactly two numeric values), or categorical (string/nominal labels). Subsequently, different pairs were subjected to appropriate statistical tests within scipy.stats (from SciPy [21]), including chi2_contingency, pearsonr, pointbiserialr, and kruskal. If pairs had fewer than five complete observations, they were excluded from the analyses, and all *p*‐values were adjusted for multiple sample comparisons using Benjamini–Hochberg false discovery rate (FDR) correction, retaining associations with an adjusted *p*‐values (*q*) ≤ 0.05. This was done using multiple tests within statsmodels.stats.multitest (from statsmodels; github.com/statsmodels/statsmodels). For completeness, all collected metadata variables were included in this cross‐association analysis, which includes pairs with known definitional dependency. These are explicitly flagged in Table S4. Of note, confounding factors, such as the country of sampling, cannot be fully excluded given the unbalanced sampling across European countries. Effect sizes for Pearson and phi are presented as PCC and phi ranging from −1 to 1, indicating the direction and strength of the statistical correlation (PCC and phi ≥ 0.40, representing moderate to very strong effect sizes). Cramér’s *V* effect sizes range from 0 to 1 and were used to assess the strength of association (*V* ≥ 0.50 considered a large effect size). All cross‐associations are summarized in Table S4. Throughout all tables and figures, HA/NA clades represent global swIAV (sub)clade classifications as obtained from NCBI/BV‐BRC, whereas HA/NA segments represent swIAV lineages (i.e., pdm09, Eurasian[EA], Scot94, Malay04, Gent84, N2Hu, and It‐N2).

## 3. Results

### 3.1. H1avN1, H1huN2, H1avN2, and H1pdmN1 swIAV Subtypes Dominate European Pig Farms

Of the 554 pooled samples, 419 (75.6%) were swIAV‐positive, corresponding to 124/134 farms (93%) among the targeted farms included in this study. The distribution of swIAV‐positive samples varied among age groups and geographic regions (Figure 2A and Table S1). Subtyping was successful in 108 farms, with most showing a single subtype (mainly H1avN1, H1avN2, H1huN2, or H1pdmN1). Cocirculation of multiple subtypes was detected in 11% of the 108 farms with successful subtyping (Figure 2B, burgundy).

**Figure 2:**
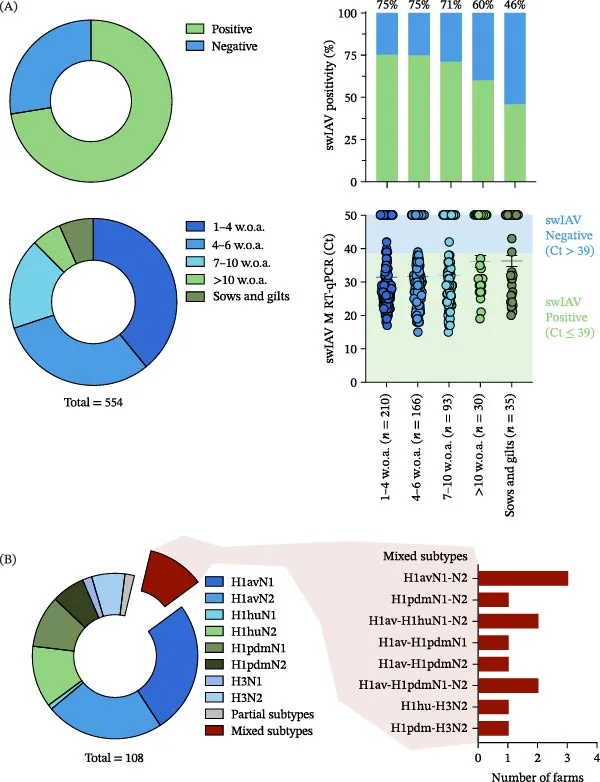
Overview of swIAV positivity and subtypes across samples based on multiplex RT‐qPCR. (A) Overall number of positive samples across the studied age groups, including age‐group specific positivity and range of associated Ct values. Samples reported as negative are visualized with a fixed Ct 50. Statistical differences were determined using a nonparametric Kruskal–Wallis test with Dunn’s multiple comparisons; no significance was identified between the age‐group specific Ct. (B) swIAV subtyping based on multiplex RT‐qPCR, highlighting HA/NA subtypes across all tested farms (*n* = 108). The mixed subtype group (*n* = 12 farms; burgundy) was further analyzed to highlight the prevalence of cocirculating strains.

### 3.2. At Least 15 Different swIAV (Sub)clades Circulate on European Farms

Most samples with RT‐qPCR positivity Ct ≤ 30 (*n* = 251) were further examined by WGS. Importantly, prolonged storage of original samples resulted in a significant loss of swIAV RNA detection, as confirmed by RT‐qPCR retesting prior to long‐read sequencing (original Ct 24.3 ± 4.0; retested Ct 26.5 ± 3.6: Figure 3A). Failure of swIAV cDNA generation and subsequent sequencing was observed in 26% of the selected samples, characterized by a higher average retested Ct (30.1 ± 4.3) as compared to 26.0 ± 3.3 for successfully sequenced samples (i.e., at least one segment; Figure 3A). The HA and NA (sub)clades could be successfully determined for 178 and 182 samples, respectively, covering a total of 92 farms (Figure 3B). In 12 farms, swIAV from different HA clades was detected, reconfirming the findings of the multiplex RT‐qPCR (Figure 2B). In three cases, different (sub)clades were observed within the same HA type (H1av), whereas in the remaining cases, multiple HA subtypes were identified. Interestingly, one Polish farm showed the cocirculation of at least three swIAV strains, representing reassorted swIAV with mixtures of 1A.3.3.2 and 1C.2.5 HA (sub)clades and N1/N2 NA lineages including Eurasian avian‐like H1avN1av (EA), A/swine/Gent/1/1984‐like H3huN2hu (Gent84), and H1pdmN1pdm (pdm09) (Table S3). As summarized in Figure 3B, global HA (sub)clade classification revealed the identification of 15 different (sub)clades across the 92 sampled farms. A dominance of the 1A.3.3.2 (H1pdm subtype; 23%) subclade was observed, followed by 1C.2.2 (19%), 1C.2.4 (16%), and 1C.2.1 (12%), all belonging to the H1av subtype.

**Figure 3:**
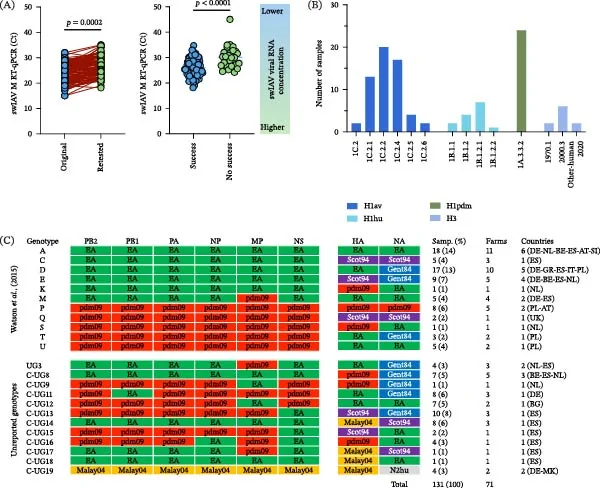
Impact of prolonged storage on Ct and swIAV sequencing success and swIAV genotyping from different European countries detected on 92 farms in the period 2022–2023. (A) Impact of prolonged storage on swIAV RNA quantification of original samples as determined by RT‐qPCR on 116 paired samples, along with retested Ct analyses on successful WGS samples (i.e., at least one segment sequenced) as compared to nonsuccessful samples. (B) In‐depth sequence analysis for global HA (sub)clades according to BV‐BRC (Bacterial and Viral Bioinformatics Resources Center or NCBI) for all unique samples across 92 European farms (*n* = 106). (Sub)clades were grouped and colored based on their subtypes—H1av (dark blue), H1hu (light blue), H1pdm (green), and H3 (light purple). (C) Genotype classification of WGS data in accordance with Watson et al. [22]. The origin of each gene segment is color coded according to their lineage (green: EA, Eurasian avian‐like H1avN1av; red: pdm09, H1pdmN1pdm; purple: Scot94, A/swine/Scotland/410440/1994‐like H1huN2hu; blue: Gent84, A/swine/Gent/1/1984‐like H3huN2hu; yellow: Malay04, A/Malaysia/29930/2004 H3huN2hu; and gray: N2hu, Human seasonal‐like N2) with known and unreported genotypes indicated as “UG” or “C‐UG.” For each of the identified genotypes, total number of samples and percentages (%) are indicated, along with the number of farms and countries they were identified in. Countries were summarized using their two‐letter code abbreviations. Abbreviations: AT, Austria; BE, Belgium; BG, Bulgaria; DE, Germany; ES, Spain; GR, Greece; HA, hemagglutinin; IT, Italy; MK, North Macedonia; MP, matrix protein; NA, neuraminidase; NL, the Netherlands; NP, nucleoprotein; NS, non‐structural; PA, polymerase acidic; PB1, polymerase basic 1; PB2, polymerase basic 2; PL, Poland; SI, Slovenia; UK, United Kingdom.

### 3.3. Unreported swIAV Genotypes Unravel swIAV Reassortment Dynamics

A total of 137 complete (i.e., all segments successfully recovered and classified) swIAV genomes from 92 farms were obtained. Of these, 131 originated from nonmixed populations, supporting accurate swIAV genotyping based on the scheme by Watson et al. [22] (Figure 3C). New genotypes were classified as “unreported genotypes” (UGs) or “Ceva UGs” (C‐UGs). This analysis revealed the circulation of a minimum of 23 distinct swIAV genotypes in European pig populations during 2022–2023. A total of 11 known and 12 new unreported swIAV genotypes were identified. The internal gene segments of 22 genotypes fully originating from Eurasian avian‐like (EA; green) and/or pdm09 (H1pdmN1pdm; red) lineages, whereas one lineage (C‐UG19) harbored all internal segments belonging to the Malay04 lineage (A/Malaysia/29930/2004 H3huN2hu; yellow). In addition, four different HA lineages (EA, A/swine/Scotland/410440/1994‐like H1huN2hu (Scot94), pdm09, and Malay04) were identified in various combinations with either one of four NA lineages (EA, Scot94, Gent84, or human seasonal‐like N2 (N2hu)) across the genotypes (Figure 3C). Of note, one partial swIAV genome from IT also showed the presence of A/swine/IT/4675/2003‐like N2 in combination with Scot94‐lineage HA and full EA lineage internal segments, with the absence of the PA segment from sequencing (Table S3). While the most prevalent and known genotypes A, D, E, and P were identified across various farms in two to six different countries, most UGs were restricted to one or two countries, except for genotype C‐UG8, which was found in BE, NL, and ES on a total of five farms (Figure 3C). Spain also showed the widest diversity of swIAV (i.e., 13 out of 23 genotypes).

### 3.4. Cross‐Association Analysis of swIAV Clades With Clinical and Management Metadata

Next, a cross‐association analysis was performed to assess statistical associations between swIAV HA/NA (sub)clades and clinical and management metadata. This allows the identification of associations within multidimensional datasets (Table S4) and the association of genotypes with clinical data and other parameters. Of note, to allow proper statistical analyses, subsampling of the data was required to prevent oversampling of a single farm. This was done using the criteria described in the methods. This resulted in the retention of 92 of the 137 complete swIAV sequences. Following removal of the id variable, a total of 4371 pairwise comparisons were conducted across 94 metadata variables, of which 442 were significant after the Benjamini–Hochberg FDR correction. With a focus on clinical signs, Figure 4A (orange circles) shows an overview of the linkage of the available clinical metadata with HA/NA (sub)clades across age groups. Overall, more data were recorded within suckling piglets and the early–mid–end nursery period across the different farms and countries (methods). This is highlighted by higher numbers of closed circles in these age groups as compared to samples from fattening (>10 w.o.a) and sows/gilts.

**Figure 4:**
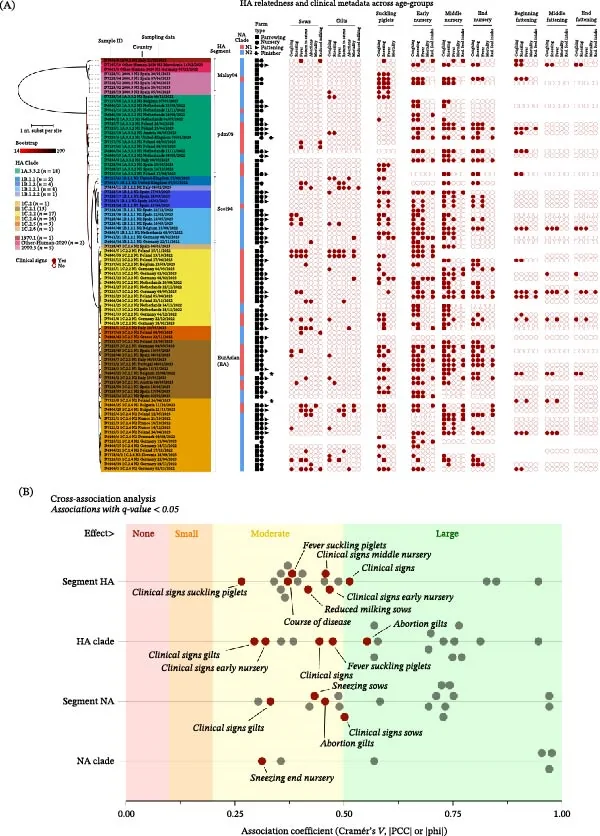
Cross‐association analysis for HA (sub)clades and clinical metadata in 92 farms on subsampled (*n* = 92/178) HA sequences. (A) Phylogenetic inference of HA sequences linked to their associated metadata. Each sequence is colored based on its HA clade and mapped to respective N1/N2 NA clades as indicated in red or blue boxes, respectively. Farm types are indicated with differential black shapes, followed by metadata on clinical signs across different age groups, represented as observed (yes; closed circle) or not observed (no; open circle). Absence of symbols indicates no data were available for this age group. (B) Focus cross‐association analysis for HA/NA segment (i.e., swIAV lineages) and (sub)clades in relation to significant (*q*‐value < 0.05) associations represented as association coefficients. Shades of red to green indicate no, small, moderate, and large effect sizes, respectively. Unannotated associations belonged mostly to significant associations within and between HA/NA segments and (sub)clades themselves. All cross‐associations can be found in Table S4.

Overall, the HA segments (i.e., swIAV lineages) showed significant (Benjamini–Hochberg FDR < 0.05) associations with the sampled animal’s clinical signs (*V* = 0.51; *q* < 0.001). In addition, age‐level clinical signs were also significantly associated with the HA segments, including early nursery (*V* = 0.47; *q* <0.001), middle nursery (*V* = 0.46; *q* < 0.001), and suckling piglets (*V* = 0.27; *q* = 0.031) (Figure 4B and Table S4). The HA (sub)clades, on the other hand, were associated with abortions (*V* = 0.55; *q* = 0.003) and clinical signs (*V* = 0.30; *q* < 0.001) in gilts and with fever in suckling piglets (*V* = 0.48; *q* = 0.024). While clinical signs of sows showed a strong association with return to estrus (*V* = 0.82; *q* < 0.001), abortions (*V* = 0.66; *q* < 0.001), and mortality (*V* = 0.62; *q* < 0.001) of gilts, clinical signs in the middle of the nursery were associated with reduced feed intake at the end of the nursery (*V* = 0.81; *q* < 0.001). Noteworthy, the internal PB1, PB2, NP, MP, and NS segments were associated with clinical signs at the end of the fattening period with significant correlation effect sizes ranging between 0.41 and 0.54. The PB1 segment was also associated with sneezing at the end of the fattening period (*V* = 0.54 and *q* = 0.011). Importantly, many other significant associations are summarized in Table S4, which are a result of associations within and between (closely) linked parameters (e.g., HA/NA segments and (sub)clades) in the cross‐tabulation.

Even though the metadata includes data on flu vaccination (i.e., type and protocol), medication (e.g., pain killers and antimicrobials), and PRRSV status and vaccination, amongst others, none of these parameters showed significant and meaningful associations with HA/NA segments or (sub)clades (Tables S3 and S4).

## 4. Discussion

This study provides a comprehensive overview of circulating swIAV subtypes and their genetic diversity across European pig farms between October 2022 and September 2023. By combining extensive molecular diagnostics and detailed metadata collection, we were able to explore potential associations between viral genotypes, clinical manifestations, and herd‐level management practices under field conditions. These findings have implications beyond veterinary health. Because swIAV lineages have repeatedly contributed to zoonotic infections and past human pandemic viruses, understanding their evolution in pig populations is essential for zoonotic risk assessment and One Health preparedness [23].

Importantly, our data reconfirmed the impact of sample quality and storage conditions and emphasized the importance of Ct cut‐offs when applying downstream long‐read nanopore sequencing. WGS was successful (i.e., at least one swIAV segment sequence was obtained), with Ct 26.2 ± 3.3. This is in line with previous reports (i.e., Ct < 27) on WGS and is an important consideration when using nanopore sequencing [24]. Sequencing enabled the identification of 23 distinct genotypes, including 12 previously unreported ones, underlining the dynamic evolution of swIAV. Fifteen different HA (sub)clades were found, often cocirculating within the same farm. These findings extend earlier surveillance reports and demonstrate that coinfections with genetically distinct strains are not isolated events but a recurring feature of commercial pig production [4, 6, 9, 20, 25–27].

Such coinfections sustain the risk of reassortment and the emergence of novel gene constellations with potentially altered virulence, host range, or transmissibility. Our data provide direct field‐based evidence that the reassortment potential is active and relevant in routine production settings. Continuous diversification of swIAV within intensive farming systems maintains a reservoir of genetically distinct viruses with varying potential for transmission and disease expression. Strengthening coordinated surveillance efforts across pig populations in Europe will be essential to improve early detection and control of evolving strains and to assess potential zoonotic implications within a broader One Health context. In this context, the particularly high genotype diversity observed in ES, one of Europe’s most intensive swine‐producing countries, is consistent with the hypothesis that production intensity and continuous pig flow may promote viral diversity and reassortment. However, it is important to note that ES was also the country with the second highest number of samples (*n* = 94) besides PL with 123 samples. A lower swIAV genotypic diversity was observed across these Polish farms.

A central aim of the study was to determine whether certain swIAV genotypes were associated with clinical outcomes. While experimental infection trials have provided valuable insights into the clinical manifestation and pathogenesis of swIAV, including lesions, shedding patterns, viral kinetics, and immune responses [28–36], these studies are typically conducted under controlled conditions with specific pathogen‐free animals, standardized viral inoculum, and short observation periods. As such, their findings cannot be directly extrapolated to field settings, where swIAV infections occur in genetically heterogeneous pig populations [37–40] under variable environmental and management conditions [41, 42]; often in the presence of maternal antibodies [43–45], concurrent pathogens, and additional stressors. Our study therefore fills an important knowledge gap by providing field‐based exploratory evidence for associations between HA/NA (sub)clades and clinical disease patterns in commercial pig herds. Although causal relationships cannot be confirmed from cross‐sectional data, understanding genotype‐phenotype patterns under field conditions is essential for assessing both disease expression and potential zoonotic risk. Even subclinical infections can sustain viral replication and reassortment, creating opportunities for the emergence of strains with human infection potential. The associations observed here, therefore, provide a valuable starting point for targeted experimental validation. For instance, HA (sub)clades showed significant associations with clinical signs as well as with abortions in gilts, highlighting clinically meaningful outcomes connecting viral genotypes to reproductive failure at the herd level. Interestingly, HA (sub)clades were also associated with fever in suckling piglets. However, it remains unclear whether these associations result from differential pathogenicity, replication kinetics, tissue tropism, or host immune interactions. Further in vivo studies using representative isolates from specific (sub)clades are warranted to elucidate the underlying mechanisms and reconfirm the observed associations. Nonetheless, these observations suggest that genetic surveillance of HA/NA (sub)clades could serve as an early indicator of clinical patterns, supporting herd‐level interventions and evidence‐based decision‐making in swine health management.

Notably, the internal gene segments PB1, PB2, NP, MP, and NS were significantly associated with clinical signs at the end of the fattening period. The association between these internal segments and clinical signs in fattening pigs, the immunologically most primed animals, suggests that whole genome swIAV segment constellations are important as they track with fattening‐stage disease beyond the HA/NA genotypes. Previous studies have shown that internal gene cassettes and reassortment patterns influence viral fitness, transmission efficiency, host adaptation, and virulence in swIAVs [46–48]. This finding further emphasizes the added value of WGS in swIAV surveillance. No significant associations were found with vaccination status, PRRSV, or antimicrobial use. Sow vaccination can reduce disease and shedding, and maternal antibodies protect piglets from clinical signs, but neither prevent infection of piglets, allowing ongoing circulation [35, 44, 49]. Experimental studies have shown that vaccination lowers the reassortment risk but does not limit viral diversification [50]. The selective pressure imposed by vaccination is both limited and variable, particularly in the presence of antigenic mismatch and further diluted by variability in vaccine composition, timing, and coverage. Moreover, in all examined farms, vaccination was limited to sows, consistent with the prevailing practice in most vaccinating herds, thereby protecting only a small proportion of the overall population. The absence of detectable associations may also reflect dataset subsampling, inherent heterogeneity, and confounding factors, which were not accounted for here. Larger, standardized longitudinal datasets across multiple countries would increase statistical power and expand the applicability of this extended cross‐association approach [20]. Additional limitations should also be acknowledged: (i) the cross‐sectional design capturing only a single time point per farm, limiting conclusions about temporal dynamics or causality, (ii) the exclusion of mixed infections or incomplete genomes to ensure statistical validity, possibly masking additional associations, (iii) the lack of serological testing to confirm subclinical infections, limiting insights into the true infection dynamics in herds without overt clinical signs, (iv) no correction for confounding factors was applied, which is exemplified for the significant association between HA (sub)clades and the country of sampling (*V* = 0.39, *q* < 0.001), confirming that causal inference is not possible from this cross‐sectional dataset, and (v) the sampling strategy focused on farms with a higher likelihood of infection, meaning that the results are not representative of the overall swine population and should be interpreted accordingly. Still, a major strength of this study is its broad geographic coverage and its extensive sample size. The use of WGS provides high‐resolution data to study reassortment dynamics, while linking these findings to clinical metadata offers unique field‐based insights.

## 5. Conclusions

This study provides a comprehensive overview of swIAV diversity across a geographically broad, targeted study population of European pig farms. A high detection rate of swIAV was observed within the selected farms, accompanied by considerable genetic diversity and ongoing reassortment. By linking viral genomic data with clinical observations and herd‐level management information, this study offers exploratory insights into potential associations between viral genotypes and field observations, warranting further investigation. Overall, the results underscore the importance of continued, harmonized genomic surveillance and highlight the potential of integrating genomic approaches into routine diagnostic and monitoring systems to support herd health management and disease control.

## Author Contributions

Conceptualization: Kathrin Lillie‐Jaschniski and Gwenaëlle Dauphin. Methodology: Zalán Homonnay, István Kiss, Julia Stadler, and Nick Vereecke. Formal analysis: Nick Vereecke. Investigation: Zalán Homonnay and István Kiss. Resources: Gwenaëlle Dauphin and Kathrin Lillie‐Jaschniski. Data curation and writing – original draft: Nick Vereecke, Julia Stadler, and Susanne Zoels. Writing – review and editing: Julia Stadler, Nick Vereecke, István Kiss, Zalán Homonnay, Kathrin Lillie‐Jaschniski, and Gwenaëlle Dauphin. Visualization: Nick Vereecke and Julia Stadler. Supervision: Julia Stadler. Project administration: Kathrin Lillie‐Jaschniski.

## Funding

Most of this work was conducted under the BioFluARN project, co‐funded by Ceva Santé Animale and the French government in the framework of the recovery plan and investment program for the future. Nick Vereecke was funded by the Flemish Agency for Innovation and Entrepreneurship (Baekeland mandate HBC.2020.2889).

## Disclosure

All authors have read and agreed to the published version of the manuscript. The funders played no role in the study design, data collection and interpretation, or the decision to submit the work for publication. This study did not use or evaluate any commercial product.

## Ethics Statement

The study protocol was reviewed and approved by the Clinical and Field Animal Ethics Committee (CFAEC) under reference number CFAEC‐2022‐11. The study involved the collection of nasal swab samples from pigs under commercial field conditions. No experimental infections or treatments were performed. All sampling procedures were conducted in accordance with the approved protocol and with the consent of the farm owners.

## Conflicts of Interest

Nick Vereecke is a former employe at PathoSense BV and Kathrin Lillie‐Jaschniski is an employe at Ceva. The other authors declare no conflicts of interest.

## Supporting Information

Additional supporting information can be found online in the Supporting Information section.

## Supporting information


**Supporting Information** Table S1: Detection of IAV by RT‐qPCR on sample level in the different countries and age groups in the 124 RT‐qPCR positive farms. w.o.a., weeks of age;  ^∗^ = Austria, Bulgaria, Cyprus, Greece, Macedonia, and Slovenia. Table S2: Standardized farm‐level metadata collected for all participating farms, including herd infection status (epidemic or endemic), farm size categories, influenza vaccination status, clinical signs of sampled animals, and production and management parameters assessed by questionnaire. Table S3: Down sampled farm‐level metadata used for cross‐association analyses. Table S4: Output of cross‐association analyses showing all pairwise associations including the performed statistical tests, *p*‐values, and Benjamini–Hochberg FDR correction, highlighting adjusted *p*‐values (*q*) with significance determined as *q* ≤ 0.05. Parameters included clinical variables, vaccination and management parameters, and viral genomic features. Table S5: Overview of clinical scoring criteria and production or reproductive outcomes recorded for different pig age groups. General clinical signs (sneezing, coughing, and fever) were scored on a semi‐quantitative scale (0 = absent, 1 = mild, 2 = moderate, and 3 = severe). Reproductive and production outcomes were recorded as the percentage of affected animals per group. Reduced feed intake was recorded as a binary variable (0 = absent, 1 = present).

## Data Availability

Data are available in the article Supporting Information.
